# Phytohormones and induction of plant-stress tolerance and defense genes by seed and foliar inoculation with *Azospirillum brasilense* cells and metabolites promote maize growth

**DOI:** 10.1186/s13568-017-0453-7

**Published:** 2017-07-17

**Authors:** Josiane Fukami, Francisco Javier Ollero, Manuel Megías, Mariangela Hungria

**Affiliations:** 1 Embrapa Soja, C.P. 231, Londrina, Paraná 86001-970 Brazil; 2 Dept. Biochemistry and Biotechnology, Universidade Estadual de Londrina (UEL), C.P. 60001, Londrina, Paraná 86051-990 Brazil; 3 Departamento de Microbiología, Facultad de Biología, Universidad de Sevilla, C.P. 41012 Seville, Spain

**Keywords:** *Azospirillum brasilense*, Leaf spray, Oxidative stress, Induced systemic resistance, *Zea mays* L.

## Abstract

*Azospirillum* spp. are plant-growth-promoting bacteria used worldwide as inoculants for a variety of crops. Among the beneficial mechanisms associated with *Azospirillum* inoculation, emphasis has been given to the biological nitrogen fixation process and to the synthesis of phytohormones. In Brazil, the application of inoculants containing *A. brasilense* strains Ab-V5 and Ab-V6 to cereals is exponentially growing and in this study we investigated the effects of maize inoculation with these two strains applied on seeds or by leaf spray at the V2.5 stage growth—a strategy to relieve incompatibility with pesticides used for seed treatment. We also investigate the effects of spraying the metabolites of these two strains at V2.5. Maize growth was promoted by the inoculation of bacteria and their metabolites. When applied via foliar spray, although *A. brasilense* survival on leaves was confirmed by confocal microscopy and cell recovery, few cells were detected after 24 h, indicating that the effects of bacterial leaf spray might also be related to their metabolites. The major molecules detected in the supernatants of both strains were indole-3-acetic acid, indole-3-ethanol, indole-3-lactic acid and salicylic acid. RT-PCR of genes related to oxidative stress (*APX1*, *APX2*, *CAT1*, *SOD2*, *SOD4*) and plant defense (pathogenesis-related *PR1, prp2* and *prp4*) was evaluated on maize leaves and roots. Differences were observed according to the gene, plant tissue, strain and method of application, but, in general, inoculation with *Azospirillum* resulted in up-regulation of oxidative stress genes in leaves and down-regulation in roots; contrarily, in general, *PR* genes were down-regulated in leaves and up-regulated in roots. Emphasis should be given to the application of metabolites, especially of Ab-V5 + Ab-V6 that in general resulted in the highest up-regulation of oxidative-stress and *PR* genes both in leaves and in roots. We hypothesize that the benefits of inoculation of *Azospirillum* on seeds or by leaf spray, as well as of leaf spraying of *Azospirillum* metabolites, are strongly correlated with the synthesis of phytohormones and by eliciting genes related to plant-stress tolerance and defense against pathogens.

## Introduction

Inoculation with *Azospirillum* spp. has been the subject of several studies (Bashan and Holguin 1998) due to their remarkable capacity of promoting growth of important cereals, i.e. maize (*Zea mays* L.), wheat (*Triticum aestivum* L.) and rice (*Oryza sativa* L.), in addition to several grasses (e.g. Hungria et al. 2010, 2016; Cassán et al. 2015; Pereg et al. 2016). The benefits in plant growth have been attributed to a variety of single or combined mechanisms that act either accumulatively or in cascade (Bashan and de-Bashan 2010), including: enhanced uptake of nutrients and water (Ardakani et al. 2011); production and secretion of phytohormones and other signaling molecules such as auxins (Spaepen and Vanderleyden 2015), cytokinins (Tien et al. 1979), gibberellins (Bottini et al. 1989) and salicylic acid (Sahoo et al. 2014); biological nitrogen fixation (Marques et al. 2017); and phosphate solubilization (Rodriguez et al. 2004). However, although *Azospirillum* spp. seem remarkable in their apparent lack of specificity in promoting growth of practically every plant genus and species investigated so far (Pereg et al. 2016), there are also indications that species and strains may vary in determinants of niche-specific adaptation to the rhizosphere that affect plant–microbe interactions (Wisniewski-Dyé et al. 2012). Examples of determinants of adaptation include reactive oxygen species (ROS) as shown with *A. lipoferum* strain 4B in the rice rhizosphere (Drogue et al. 2014). ROS molecules encompass free radicals resulting from oxygen metabolism such as superoxide radicals (O_2_^−^), hydroxyl radicals (OH^−^), hydrogen peroxide (H_2_O_2_) and singlet oxygen (^1^O_2_) (Bowler et al. 1992; Gill and Tuteja 2010). The most important ROS detoxification mechanism is represented by the activity of superoxide dismutase (*SOD*), ascorbate peroxidase (*APX*) and catalase (*CAT)* enzymes responsible for the scavenging of H_2_O_2_ by its conversion to water and O_2_ (Lamb and Dixon 1997; Asada 1999). In general, ROS detoxification systems vary with plant species, cultivar, and age, and also with the type and duration of abiotic and biotic stress (Hodges et al. 1996).

Another intriguing feature of *Azospirillum* spp. is that although the species comprise non-pathogenic bacteria, they are also able to induce plant-defense mechanisms that may help against further pathogen attacks (Cassán et al. 2014). This property is called ‘induced systemic resistance’ (ISR), in which the bacterium triggers a plant reaction by emitting signals—the pathogenesis-related proteins (PRs)—that spread systemically throughout the plant and enhance the defensive capacity of distant tissues against infection by pathogens (Van Loon and Bakker 2005). Once induced, plants may remain protected for prolonged periods (Van Loon 2007). For example, there are reports of *Azospirillum* helping protection against *Colletotrichum acutatum* (anthracnose) in strawberry (*Fragaria ananassa* Duch.) (Tortora et al. 2011), and resistance to *Clavibacter michiganensis* subsp. *michiganensis* (bacterial canker), *Xanthomonas campestris* pv. *vesicatoria* (Romero et al. 2003) and *Rhizoctonia solani* (damping-off disease) (Gupta et al. 1995) in tomato plants (*Lycopersicon esculentum* Mill).

Reports of plant-growth improvement by the exogenous application of synthetic growth regulators (e.g. auxins, gibberellins, cytokinins) have long been the subject of studies (e.g. Halmann 1990); more recently, emphasis has also been given to their effect in increasing tolerance of abiotic and biotic stresses (Robert-Seilaniantz et al. 2011). Similar effects on stresses have been reported with the application of jasmonic acid (Bari and Jones 2009; Wasternack 2007; Lorenzo and Solano 2005) and salicylic acid (Bari and Jones 2009), which might induce *PR* (pathogenesis-related) genes and, consequently, enhance resistance to several pathogens.

The commercial use of *Azospirillum brasilense* strains Ab-V5 and Ab-V6 on maize (*Z. mays* L.) and wheat (*T. aestivum* L.) crops in Brazil has grown exponentially since 2010 (Hungria et al. 2010; Hungria 2011). Our research group has started to investigate the effects of foliar-spray inoculation of *Azospirillum*, with the main practical purpose of avoiding the contact of the bacteria with harmful pesticides that are heavily applied to the seeds (Fukami et al. 2016). In this study we confirmed benefits to plant growth by seed and foliar applications of *Azospirillum*, but also verified responses to the application of their metabolites. We then investigated phytohormone production and the response of antioxidant systems with different methods of application of *Azospirillum* strains and their metabolites.

## Materials and methods

### Bacterial strains and inoculation methods

Bacteria consisted of strains Ab-V5 (=CNPSo 2083) and Ab-V6 (=CNPSo 2084) of *Azospirillum brasilense* (from the “Culture Collection of Diazotrophic and Plant Growth-Promoting Bacteria of Embrapa Soja”, WFCC # 1213, WDCM # 1054). Both strains were derived from an *Azospirillum* selection program (Hungria et al. 2010) and are currently employed in commercial inoculants in Brazil (Hungria 2011).

The inoculants were initially prepared in DYGS medium (Rodrigues Neto et al. 1986) and, after growth for 48 h, cell concentrations were adjusted to 10^8^ mL^−1^. For the production of metabolites, inoculants were produced under the same conditions and up to the same concentration and were centrifuged at 5000 rpm for 15 min. By plating the supernatants obtained on DYGS medium we confirmed that they were free of *Azospirillum* cells.

Three methods of inoculation were compared: (i) standard seed inoculation (SI)—considered as the control; (ii) inoculation by leaf spray (ILS) at the V2.5 stage of the maize growth cycle (Hickman and Shroyer 1994); and (iii) application with metabolites from *A. brasilense* strains Ab-V5 and Ab-V6 by leaf spray (MLS) at the V2.5 stage (about 7 days after transplanting) (Hickman and Shroyer 1994).

Seeds were inoculated 1 h before sowing by thoroughly coating them to provide a final concentration of 1.6 × 10^5^ cells seed^−1^. For leaf-spray inoculation, an aerograph atomizer was employed to mimic the action of a spraying apparatus. The soil surface was covered with aluminum foil to prevent the inoculant reaching it. The final volume of liquid for leaf-spray inoculation was 1 mL (water + inoculant) per pot containing a single plant, and inoculants were diluted with sterile distilled water at 1:1000 (v:v) for spraying, to achieve an application rate of 1.6 × 10^5^ cells plant^−1^. For leaf spray of metabolites, bacterial exudate corresponding to the same cell concentration as the seed inoculant used for leaf spray was used, with the application of 1 mL per plant corresponding to 1.6 × 10^5^ cells plant^−1^. Foliar-spray inoculations of pots containing maize plants were performed 7 days after transplanting.

### Greenhouse experiment

The experiment was performed under greenhouse conditions, using modified Leonard jars (Vincent 1970) containing sterilized substrate, consisting of a mixture of sand and pulverized coal (3:1, v/v) with application of sterile nutrient solution (Fahraeus 1957). Jars were arranged in a completely randomized design with nine treatments, a non-inoculated control, and six replicates. Each treatment received 60 kg N ha^−1^ (50% of the N application recommended for the crop). Inoculation treatments consisted of mineral-N fertilizer (50% N) and different methods of inoculation: SI (standard seed inoculation at sowing), ILS (inoculation by leaf spray, at the V2.5 stage of maize growth) and MLS (inoculation with metabolites by leaf spray of *A. brasilense* strains Ab-V5 and Ab-V6 at the at the V2.5 stage).

Hybrid maize seeds (DKB330 VT PRO2) were surface-sterilized with 70% ethanol and 3% sodium hypochlorite (Vincent 1970). They were pre-geminated for 48 h at 25 °C in Petri plates containing 1% (v/v) water agar. After germination, two seedlings were transplanted per jar and thinned to one plant after 3 days. Temperature at the greenhouse in controlled by means of air conditioners and average of day and night temperatures were of 28 ± 2.3/23 ± 1.9 °C (day/night); the experiment was performed at the summer growing season, where light intensity is the most adequate for maize growth. Sterile nutrient solution was applied as needed.

At 30 days after transplanting, leaf-chlorophyll contents (CC) were determined according to Kaschuk et al. (2010), based on the “Soil Plant Analysis Development” (SPAD) index, with readings taken from the lowermost third of the +3 leaf (Trani et al. 1983). Biometric parameters of plant height (cm; PH) and culm diameter (mm; CD) of plants were determined with the aid of a digital caliper. Plants were harvested, separating leaves and roots, with three biological replicates. Fresh weight was determined and 2 g of fresh material of each sample were dried at 60 °C for approximately 72 h, until constant weights were achieved; tissues were weighed to estimate the factor for conversion from fresh to dry weight of each replicate. The remaining sampled tissues were frozen in liquid nitrogen and stored at −80 °C until further analyses.

Data obtained were first evaluated for normality and variance homogeneity, followed by the analysis of variance (ANOVA). Tukey’s test was employed to compare means in cases where statistical significance had been detected by the ANOVA F test (*p* ≤ 0.05). Statistica software version 7.0 was employed.

### Identification of phytohormones produced by *A. brasilense* by UHPLC-HRMS/MS

The identification of phytohormones produced by *A. brasilense* strains Ab-V5 and Ab-V6 was performed by ultrahigh-performance liquid chromatography-high-resolution mass spectrometry (UHPLC-HRMS/MS). Strains Ab-V5 and Ab-V6 were grown separately in DYGS medium (Rodrigues Neto et al. 1986) without tryptophan (TRP) or in DYGS supplemented with 500 µg mL^−1^ tryptophan (DYGS-TRP medium). Liquid bacterial inocula were incubated at 28 ± 2 °C with orbital shaking at 120 rpm for 14 days. The bacterial cultures were then filtered through nitrocellulose-membrane filters Millipore HA 0.45 µm to obtain the supernatants. The samples were filtered again in a microfiltration membrane, and 5-µL aliquots of each sample were analyzed. Hormones were identified by mass/charge ratio (*m/z*) values and by the retention times of the standard compounds indole-3-acetic acid (IAA), indole-3-butyric acid (IBA), indole-3-ethanol (TOL), indole-3-lactic acid (ILA), indole-3-pyruvic acid (IPyA), indole-3-propionic acid (IPA), kinetin (Kin), gibberellic acid (GA3), salicylic acid (SA) and jasmonic acid (JA); tri-methyl-indole-3-acetic acid (TmIAA) was used as internal standard.

### RNA extraction, cDNA synthesis and quantitative RT-PCR

RNAs of leaves and roots were extracted with TRIzol^®^ (Life Technologies/Thermo Fisher Scientific), and the concentration and purity were evaluated in a NanoDrop^®^ ND1000 spectrophotometer (NanoDrop-Technologies, Inc.), while the integrity was evaluated by gel electrophoresis. Genomic DNA was removed with DNAseI (Invitrogen™) and the first strand of cDNA was synthesized using SuperscriptIII™ reverse transcriptase (Invitrogen™), according to the manufacturer’s protocol.

Primers for the RT-qPCR targets were designed using primer3Plus (http://www.bioinformatics.nl/cgi-bin/primer3plus/primer3plus.cgi/) (Table 1) to obtain amplicons of 110–150 bp. The endogenous control genes of maize used were UBCE and UBCP, corresponding to the ubiquitin-conjugating enzyme and the ubiquitin carrier protein, respectively (Manoli et al. 2012). These genes were used for data normalization of the cycle threshold (Ct) of RT-qPCR amplifications.

**Table 1:** Primers sequences used in the RT-qPCR analyses and sizes of the PCR products obtained

Target gene	Primer sequences (5′–3′)	Amplicon size (pb)
*CAT1*	*CAT1*F: ACAGCGATGAGTTGTGACGT	113
	*CAT1*R: ATCCTTGCTGCATCTGTCCG	
*SOD2*	SOD2F: GAGCACCTCAGGATGTTGCT	133
	*SOD2*R: CAGGTGCGCAACATTGTTCA	
*SOD4*	*SOD4*F: CGTCACCAGCAGGCTAGAAT	139
	*SOD4*R: AGCCAACAGTCCAACACAGT	
*APX1*	*APX1*F: GATCTTGTGGCTGCAGCATG	111
	*APX1*R: GGTGGACTCGAATTGCAGGA	
*APX2*	*APX2*F: ACGAAGATGTGATGAACTCAGC	138
	*APX2*R: GGCATTGGCATCGTTAATCAGT	
*PR1*	*PR1*F: ACTGCAAGCTGATCCACTCC	134
	*PR1*R: TGTTGGTGTCGTGGTCGTAG	
*prp2*	*prp2*F: ATTCATCGACGCGTCACAGT	117
	*prp2*R: CAGAGACAAGGACACGGACC	
*prp4*	*prp4*F: TACGACCACGACACCAACAG	143
	*prp4*R: GCTGCAGATGATGAAGACGC	

RT-qPCR reactions were performed in a 7500 RT-qPCR thermocycler (Applied Biosystems/Life Technologies). The reactions were performed in triplicate for each of the three biological replicates. The Platinum^®^ SYBR^®^ Green qPCR SuperMix-UDG (Invitrogen™) was used following the manufacturer’s instructions. Cycling conditions were as follows: 50 °C for 2 min, 95 °C for 10 min, 45 cycles at 95 °C for 2 min, 60 °C for 30 s and 72 °C for 30 s, in 45 cycles.

The data obtained were submitted to the Rest2009 software package (Pfaffl et al. 2002), providing a robust statistical analysis (*p* ≤ 0.05).

### Confocal laser scanning microscopy of *A. brasilense* on maize leaves

Maize leaf colonization by *A. brasilense* strains Ab-V5 and Ab-V6 expressing the *egfp* (enconding for enhanced green fluorescent protein) and *eyfp* (encoding for enhanced yellow fluorescent protein) reporter genes were analyzed by Confocal Laser-Scanning Microscopy (CLSM). First, plasmids pMP4655 (*egfp*) and pMP4658 (*eyfp*) (Bloemberg et al. 2000) were transferred by conjugation to *A. brasilense* Ab-V5 and Ab-V6. To select the transconjugants of *A. brasilense,* plates with DYGS agar medium (Rodrigues Neto et al. 1986) were supplemented with nalidixic acid (final concentration 40 μg mL^−1^) and tetracycline (final concentration 20 μg mL^−1^). The *Azospirillum* strains exhibit intrinsic resistance to the antibiotic nalidixic acid, whereas *Escherichia coli* containing the transfer plasmid shows only tetracycline resistance. Transconjugants were obtained for both strains of *Azospirillum*.

Seeds of maize (hybrid DKB330 VT PRO2) were surface-sterilized (Vincent 1970). Pre-germinated seeds (2 days) were transplanted to test tubes containing 70 mL of sterilized nutrient solution (Fahraeus 1957), and were grown under controlled greenhouse conditions. Mean temperatures during the experiment were of 25/18 °C (day/night) and relative humidity of 70%. At the V2.5 stage of maize growth, plants were singly inoculated by leaf spray with either *A. brasilense* strain Ab-V5 or Ab-V6 harboring the reporter plasmids expressing *egfp* and *eyfp* genes, respectively. Inoculant concentrations applied to the leaves were estimated at 3 × 10^5^ and 7 × 10^5^ cells cm^−2^ of leaf, for strains Ab-V5 and Ab-V6, respectively. At 1 h, 1 and 2 days after leaf spraying, the leaves were examined for the presence of fluorescent bacteria using CLSM equipped with an Ar–Hg laser (Leica TCS SP2, Leica, Wetzlar, Germany); the filter sets for fluorescence microscopy consisted of a 458-nm band-pass excitation and a 520–560 nm emission. Microscopy analyses were performed on intact alive plant tissues. Simultaneously to the analysis by microscopy, the presence of the bacteria on the leaves surface was verified by evaluation of colony-forming units evaluated by the drop plate method (Miles et al. 1938) 1 h, 1 and 2 days after leaf spraying.

## Results

### Effects of inoculation of *Azospirillum brasilense* and their metabolites on plant-growth parameters

In the greenhouse experiment performed to evaluate effects of inoculation on plant growth, it is worth mentioning that all treatments received the same amount of N-fertilizer, corresponding to 50% (60 kg of N ha^−1^) of the dose recommended for the maize crop in Brazil. When different methods (via seed—SI at sowing or by leaf spray—ILS at the V2.5 stage) of inoculation of *A. brasilense* strains Ab-V5 and Ab-V6, in single or combined mixtures, or foliar-spray application of their metabolites (MLS), also at the V2.5 stage, were evaluated, statistically significant increases in chlorophyll content (CC) in relation to the non-inoculated control were observed in all treatments except for the SI with Ab-V5; the highest increases were observed in the treatments with MLS of Ab-V6 and MLS of Ab-V5 + Ab-V6, of 109 and 143%, respectively (Table 2). No statistical differences were observed for the parameters of plant high (PH) and culm diameter (CC). Shoot dry weight (SDW) was also improved by all inoculation treatments, except for MLS of Ab-V5. The best inoculation treatment of MLS of strains Ab-V5 and Ab-V6 increased SDW by 72%. In relation to this best treatment (T10), we should mention that the effect might be attributed mainly to the metabolites of Ab-V6, as the single metabolites of Ab-V6 (T9), but not of Ab-V5 (T8), resulted in increases in SDW (Table 2).

**Table 2:** Effects of inoculation with *Azospirillum brasilense* strains Ab-V5 and Ab-V6 applied via seeds (seed inoculation, SI, at sowing) or by foliar application (inoculation by leaf spray, ILS, at the V2.5 stage) and of application of their metabolites (MLS) at the V2.5 stage on the chlorophyll content (CC), plant height (PH), culm diameter (CD) and shoot dry weight (SDW) of maize plants (DKB330 VT PRO2)

Treatment	CC (µg cm^−2^)	PH (cm)	CD (mm)	SDW (g pl^−1^)
T1: non-inoculated control	4.45 e^a^	57.33 a	12.22^ns^	3.24 c
T2: SI Ab-V5	5.03 e	57.00 a	13.46	4.56 ab
T3: SI Ab-V6	7.00 d	63.60 a	13.35	5.71 a
T4: SI Ab-V5 + Ab-V6	8.51 c	59.17 a	12.84	4.85 ab
T5: ILS Ab-V5	6.80 d	58.40 a	13.23	4.84 ab
T6: ILS Ab-V6	8.04 c	63.00 a	12.74	4.67 ab
T7: ILS Ab-V5 + Ab-V6	7.07 d	59.67 a	12.97	4.67 ab
T8: MLS Ab-V5	7.08 d	65.40 a	12.16	4.15 bc
T9: MLS Ab-V6	9.30 b	60.50 a	12.49	5.39 ab
T10: MLS Ab-V5 + Ab-V6	10.80 a	64.67 a	13.34	5.57 a
*p* value	<0.0001	0.03915	0.2609	<0.0001
CV (%)	10.11	8.50	8.69	13.81

All treatments received the equivalent of 60 kg of N ha^−1^ at sowing, plants were grown under greenhouse conditions and harvested at 30 days after transplanting

^a^Means (six replicates) followed by the same letter on the same column are not statistically different according to the Tukey’s test (*p* ≤ 0.05); ^ns^ statistically non-significant

### Identification of phytohormones produced by *A. brasilense* by UHPLC-HRMS/MS

UHPLC-HRMS/MS results obtained in the analysis of the supernatants from *Azospirillum* strains grown in DYGS or DYGS + TRP (tryptophan) media are presented in Table 3. For all samples, supplemented or not with tryptophan, the following main compounds were detected in the metabolites: indole-3-acetic acid (IAA), indole-3-ethanol (TOL), indole-3-lactic acid (ILA) and salicylic acid (SA). Other compounds have also been identified, but in relatively low amounts. In the supernatant of Ab-V5 grown without TRP, we detected gibberellic acid (GA_3_) and jasmonic acid (JA) and, when supplemented with TRP, we detected indole-3-propionic acid (IPA). In the supernatant of strain Ab-V6 supplied with TRP we detected GA_3_ (Table 3).

**Table 3:** Identification by ultrahigh-performance liquid chromatography-high-resolution mass spectrometry (UHPLC-HRMS/MS) of phytohormones produced by *A. brasilense* strains Ab-V5 and Ab-V6 after 14 days of growth on DYGS medium supplemented or not with tryptophan (TRP, 500 µg mL^−1^)

Treatment	IAA^a^	IBA	TOL	ILA	IPyA	IPA	Kin	GA_3_	JA	SA
Ab-V5	+^b^	−	+	+	−	−	−	*	*	+
Ab-V5 + TRP	+	−	+	+	−	*	−	−	−	+
Ab-V6	+	−	+	+	−	−	−	−	−	+
Ab-V6 +TRP	+	−	+	+	−	−	−	*	−	+

^a^Indole-3-acetic acid (IAA), indole-3-butyric acid (IBA), indole-3-ethanol (TOL), indole-3-lactic acid (ILA), indole-3-pyruvic (IPyA), indole-3-propionic acid (IPA), kinetin (Kin), gibberellic acid (GA_3_), jasmonic acid (JA), salicylic acid (SA)

^b^+ detected; − no detected; * low relation

### Expression of genes related to defense mechanisms in maize

**Fig. 1:**
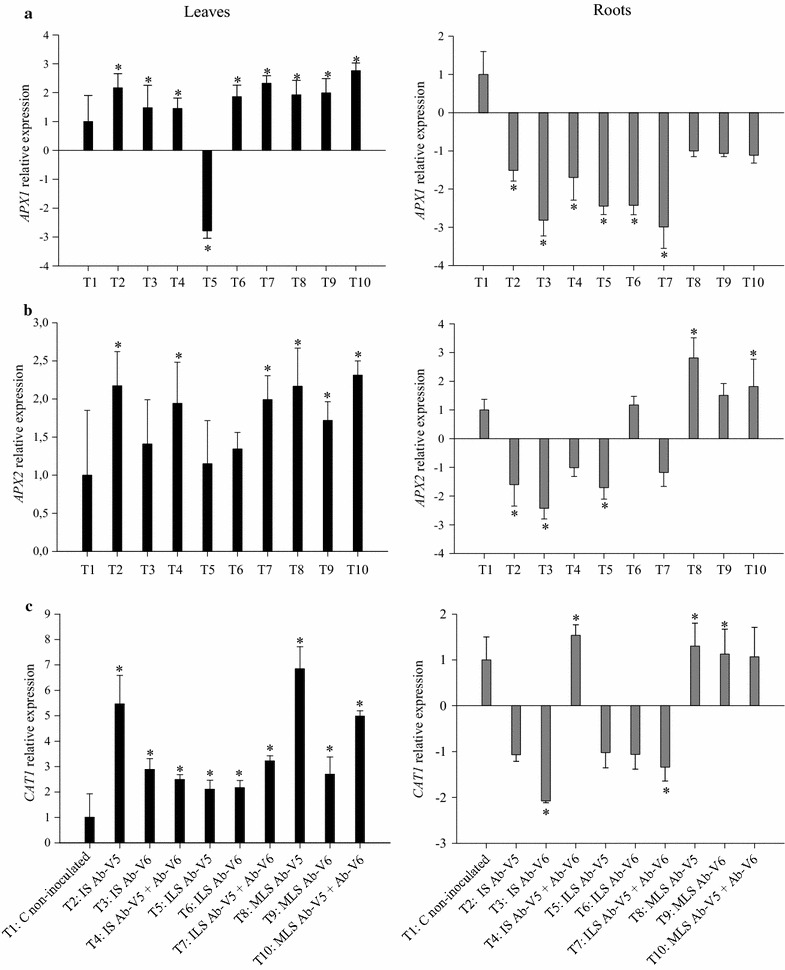
RT-qPCR analysis of the expression of **a** *APX1*, **b** *APX2* and **c** *CAT* genes in maize leaves and roots when induced by *Azospirillum brasilense* strains Ab-V5 and Ab-V6 inoculated on seeds or by foliar spray, and also buy their metabolites applied by foliar spray. Data ± standard deviation from three biological replicates, each with three technical replicates. Data were normalized in relation to the endogenous control (UBCE and UBCP). The *asterisks* indicate statistically significant expression at the level α = 5%, determined by REST2009 software. *Black bars* leaf and *dark gray bars* root

**Fig. 2:**
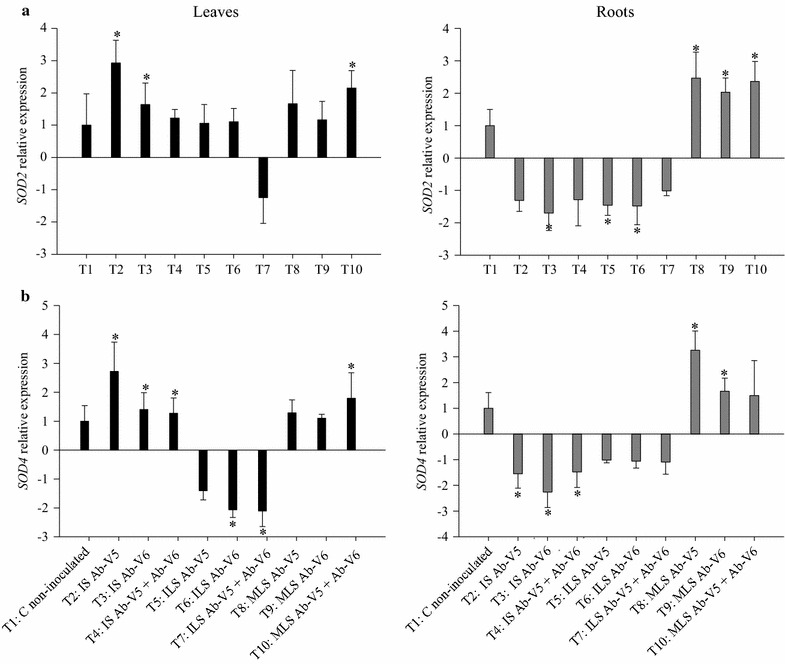
RT-qPCR analysis of the expression of **a** *SOD2* and **b** *SOD4* genes, as described in the legend of Fig. 1

**Fig. 3:**
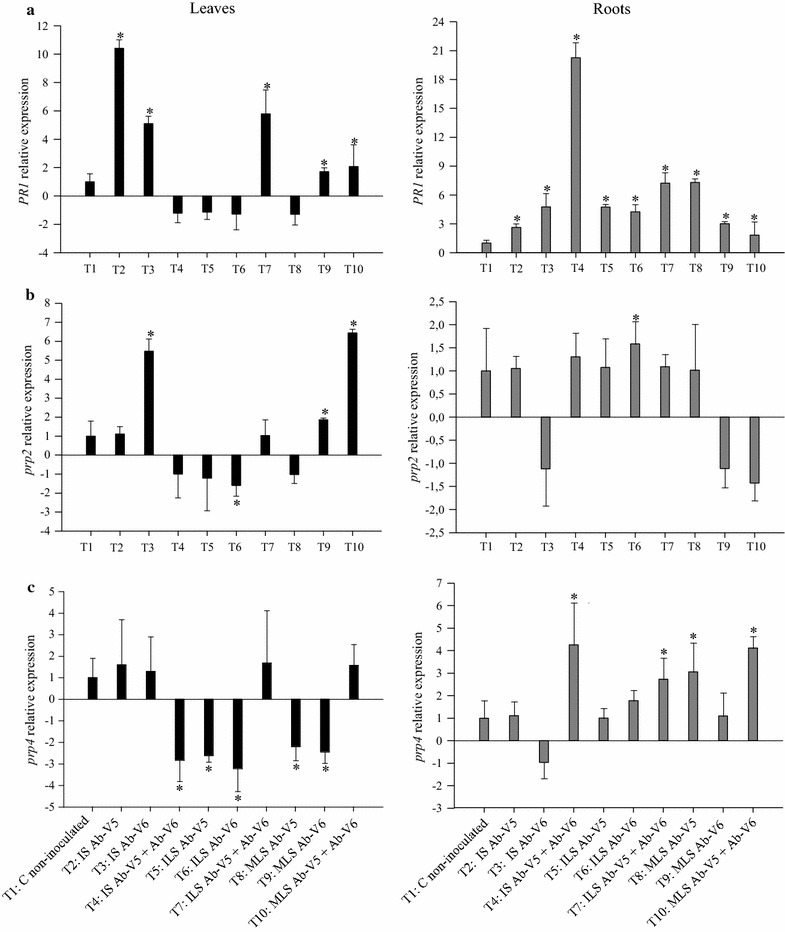
RT-qPCR analysis of the expression of several **a** *PR1*, **b** *prp2* and **c** *prp4* genes, as described in the legend of Fig. 1

Effects of inoculation with *Azospirillum* or their metabolites on the expression of genes encoding for antioxidant and *PR* proteins were determined by RT-qPCR (Figs. 1, 2, 3). When compared to the non-inoculated control (T1), the gene of the cytosolic isoform *APX1* in maize leaves was significantly up-regulated by inoculation in all treatments except for with the inoculation with strain Ab-V5 by leaf spray (T5) (Fig. 1a). The highest expression was achieved with treatment T10, with inoculation of metabolites of both strains, with an increase of 2.8-fold in comparison to the non-inoculated control. Contrarily, the expression of *APX1* in roots was down-regulated in all treatments (Fig. 1a). The expression of the *APX2* gene in leaves was up-regulated in all treatments, and statistically significant in six out of the nine inoculation treatments (Fig. 1b). Contrarily to *APX1* gene-expression in roots, *APX2* was significantly up-regulated with the metabolites of Ab-V5 (T8), and the metabolites of Ab-V5 + Ab-V6 (T10) (Fig. 1b). The same trend as for *APX* genes was observed with *CAT1* (Fig. 1c). The highest expression in leaves was achieved by seed inoculation with Ab-V5 (T2, 5.5-fold) and spraying of the metabolites of the same strain (T8, 6.9-fold). CAT1 was down-regulated in roots, except for the seed inoculation with Ab-V5 + Ab-V6 (T4) and the metabolite-spray treatments (T8, T9, T10) (Fig. 1c). When *SOD* genes were investigated, up-regulation in leaves was achieved in all treatments, except for when both strains were leaf sprayed for *SOD2* (Fig. 2a) and when *Azospirillum* cells were leaf sprayed for *SOD4* (Fig. 2b). Contrarily, both genes were down-regulated in roots when living cells were applied to seeds or sprayed, whereas the application of metabolites on leaves resulted in up-regulation, with the highest expression of 2.5-fold for *SOD2* and of 3.2-fold for *SOD4* with the metabolites of Ab-V5 (Fig. 2a, b).

Analyzing the *PR* group of genes (*PR1, prp2* and *prp4*) (Fig. 3a–c), in general seed inoculation with single strains (Ab-V5 or Ab-V6) up-regulated gene expression in leaves, whereas seed co-inoculation, and foliar inoculation with single strains down-regulated the genes. Seed inoculation with Ab-V5 (T2) increased by 10.4-fold the expression of *PR1* gene in leaves, whereas, with Ab-V6 (T3), up-regulation was of 5.1- and 5.5-fold for *PR1* and *prp2*, respectively. In relation to the effects of metabolite sprays on gene expression in leaves, emphasis should be given to the Ab-V5 + Ab-V6 treatment (T10), always showing up-regulation, in particular of *prp2* (6.4-fold). In relation to the gene expression in roots (Fig. 3a–c), in general all treatments resulted in up-regulation, but emphasis should be given to the co-inoculation of seeds on the expression of *PR1* (20.2-fold) and *prp4* (4.2-fold), respectively; down-regulation of *prp2* with the metabolites of Ab-V6 of Ab-V5 + Ab-V6 was not statistically significant (Fig. 3b).

### Colonization of maize leaves by *A. brasilense*

**Fig. 4:**
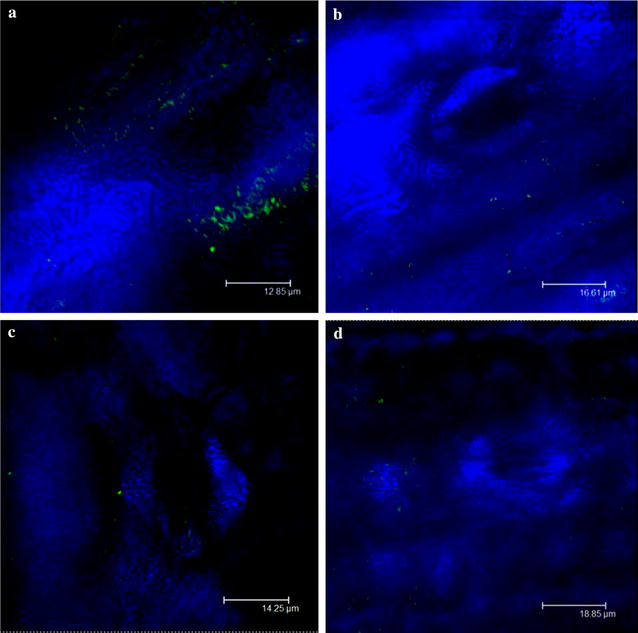
Confocal laser scanning microscopy analysis of maize leaf surface colonization by *A. brasilense* expressing EGFP when inoculated by leaf spray. **a** *A. brasilense* Ab-V5 after 1 h, **b** *A. brasilense* Ab-V5 after 1 day, **c** *A. brasilense* Ab-V6 after 1 h, **d** *A. brasilense* Ab-V6 after 1 day of inoculation

In order to check whether *A. brasilense* cells are able to colonize maize leaves, strains Ab-V5 and Ab-V6—harboring reporter plasmids expressing *egfp* and *eyfp* genes, respectively—were inoculated by leaf spray. After 1 h, 1 and 2 days of inoculation, the leaves were visualized by CLSM (Fig. 4). After 1 h of inoculation with both strains, (EGFP)-l and (EYFP)-labelled cells indicated that they were able to colonize leaves surface (Fig. 4a, d), and the same was observed after 1 day of inoculation (Fig. 4b, c). However, after 2 days of inoculation, we were unable to detect the strains on the leaf surfaces. Simultaneously, bacteria counts on leaves surface were performed after 1 h, 1 and 2 days of leaf spraying. Values obtained for colony-forming units (CFUs) were as follows: 2 × 10^5^, 1 × 10^5^ and 5 × 10^2^ CFUs cm^−2^ of leaf for strain Ab-V5 and 6 × 10^5^, 5 × 10^5^ and 5 × 10^2^ CFU cm^−2^ of leaf for strain Ab-V6 after 1 h, 1 and 2 days, respectively. The low bacterial counts at 2 days after inoculation might explain why the bacteria were not visualized by CLSM.

## Discussion

When maize growth was evaluated under greenhouse conditions, the benefits of inoculation with *A. brasilense* Ab-V5 and/or Ab-V6 applied to seeds or by foliar application at the V2.5 stage of plant growth were confirmed. The benefits of inoculation with *Azospirillum* at sowing, via seeds or in-furrow, have been demonstrated under greenhouse and field conditions in cereals, with an emphasis on maize (Dobbelaere and Okon 2007; Hungria et al. 2010; Hungria 2011; Okon et al. 2015; Fukami et al. 2016), and increasing use of strains Ab-V5 and Ab-V6 has been exponential in Brazil since 2010 (Hungria 2011). Improvements in grain yields of maize and wheat by foliar application of *Azospirillum* have also been reported (Clemente et al. 2016; Fukami et al. 2016), but the physiological and genetic basis of such improvements have yet to be elucidated.

Intriguing were the positive responses observed in our study to foliar application of metabolites of *Azospirillum*—especially with Ab-V5 + Ab-V6—at the V2.5 stage. Previously, we reported benefits to the maize crop by the application of metabolites of rhizobia, suggesting that the effects could be attributed to lipo-chitooligosaccharides (LCOs) or Nod factors (Marks et al. 2013, 2015) synthesized by the bacteria. Positive effects with application of Nod factors in maize, cotton (*Gossypium hirsutum*) and beet (*Beta vulgaris*) were also reported by Smith et al. (2015). However, as far as we are aware, this is the first scientific report of effects of *Azospirillum* metabolites on cereal growth.

To achieve a better understanding of the effects of leaf spraying with *Azospirillum* cells, we investigated the bacterial colonization of leaves by microscopy. Strains Ab-V5 and Ab-V6 were detected on leaves surfaces up to 24 h after inoculation, but the numbers of surviving cells (CFU) were markedly reduced, and, after 48 h, cells were not detected by microscopy. It is possible that the number of recovered cells after 24 h was too low to be detected by CLSM, but the mortality in 24 h was of the order of 1000-fold. Furthermore, we must bear in mind that our experiment was performed under controlled optimized conditions, and that mortality under stressful field conditions—UV light, desiccation, high temperature—would certainly be far higher. Therefore, it is reasonable to suggest that the benefits observed in our study from foliar spraying of *Azospirillum* cells resulted from metabolites present in the inoculant rather than from the living cells.

The first hypothesis to explain increased plant growth by spraying cells or metabolites of *A. brasilense* Ab-V5 and Ab-V6 relies on phytohormone production. We have identified the main molecules in the supernatants of the Ab-V5 and Ab-V6 strains, induced and non-induced with tryptophan, as being indole-3-acetic acid (IAA), indole-3-ethanol (TOL), indole-3-latic acid (ILA) and salicylic acid (SA). Although the physiological functions of TOL and ILA remain unknown, it is possible that intermediates of IAA biosynthesis pathways are converted into these storage compounds whenever necessary (Cassán et al. 2014). In addition, in some combinations of strains and tryptophan we detected traces of gibberellic acid (GA_3_) and jasmonic acid (JA). The synthesis of phytohormones by *Azospirillum* has been broadly reported, and may differ between species and strains. The well studied *A. brasilense* strains Cd and Az39 produce IAA, zeatin, GA_3_, abscisic acid and ethylene (Perrig et al. 2007), strain UAP154 produces IAA and indole-butyric acid (IBA) (Martínez-Morales et al. 2003), strain 703Ebc produces IAA, TOL, ILA and indole-3-methanol (Crozier et al. 1988), and Sp13t produces IAA, ILA, GA_3_ and kinetin (Tien et al. 1979). Tien et al. (1979) also detected gibberellin-like molecules in the supernatants of *A. brasilense* Sp13t at low concentrations, of about 0.05 µg of GA_3_ mL^−1^. However, when applied at concentrations as low as 0.005 µg mL^−1^ to lettuce (*Lactuca sativa*), hypocotyls elongation was promoted and, in pearl millet (*Pennisetum americanum* L.), the number of lateral roots was increased. The benefits confirmed in our study of inoculation of seed with *Azospirillum* at sowing may be attributed to the effects of phytohormones in the rhizosphere, and we propose that these effects also occur from the application of cells and metabolites to the leaves.

Plants synthesize a variety of secondary metabolites that are involved in several physiological processes, and main functions of these compounds lie in providing stress tolerance and defense against pathogens (Sudha and Ravishankar 2002). Previous studies have reported that maize inoculation with *Azospirillum* results in significant changes in the secondary metabolic profiles of roots and shoots, suggesting the presence of finely-tuned interacting mechanisms (Walker et al. 2011). In addition, reactive oxygen species (ROS) in plants contribute to resisting biotic stresses such as pathogens and even symbiotic bacteria (before plant perceives benefit from the symbiosis) (Lamb and Dixon 1997; Santos et al. 2001), as well as to tolerating abiotic stresses (Ozyigit et al. 2016), such as saline conditions (Barakat 2011). However, ROS accumulation results in oxidative damage to cells such as lipid peroxidation with membrane destruction, protein inactivation or DNA mutation (García-Limones et al. 2002). Oxidative stress is relieved in plants by antioxidant enzymes such as catalase, superoxide dismutase and ascorbate peroxidase (Wisniewski-Dyé et al. 2012; Ozyigit et al. 2016). The genes encoding the isoenzymes are found in different plant-cell compartments, such as the cytosolic *SOD2*, *SOD4* (Jung et al. 2001), *APX1* and *APX2*, which are inducible mainly under extreme light or heat-stress conditions (Davletova et al. 2005), and *CAT1*, found in peroxisomes, glyoxysomes and also in the cytosol (Scandalios et al. 1997; Jung et al. 2001). We evaluated the effects of *Azospirillum* and its metabolites on the expression of genes related to the synthesis of the H_2_O_2_-generating enzyme (*SOD*), the H_2_O_2_-scavenging enzymes (*CAT* and *APX*) in maize leaves and roots. In general, inoculation of seeds with *A. brasilense* and by foliar spraying resulted in down-regulation transcription of oxidative stress genes (*APX1*, *APX2*, *SOD2*, *SOD4*) in roots, but genes were always up-regulated by leaf spray of metabolites, except for *APX1*. The results suggest that oxidative stress in roots persisted longer with the application of living cells than with their metabolites. Seed inoculation up-regulated all genes in leaves, but when cells were sprayed on leaves, *SOD4* with all strains and *APX1* with Ab-V5 were down-regulated. Similarly to the roots, when the metabolites were sprayed on the leaves the genes—now including *APX1*—were up-regulated. The up-regulation of *APX1* in leaves is particularly interesting, as *APX* genes might be essential for chloroplast protection during light stress (Pnueli et al. 2003; Mittler et al. 2004; Davletova et al. 2005).

Another defense mechanism of the plants is mediated by ISR (induced systemic resistance), resulting in plant resistance to some pathogenic bacteria, viruses and fungi (Lugtenberg and Kamilova 2009). ISR is triggered by non-pathogenic microorganisms and starts in primary infected parts, extending to other plant tissues (Dutta et al. 2008). Biochemical or physiological changes in plants include induced accumulation of pathogenesis-related (PR) proteins that have different functions like the proteins encoded by *PR1* (a member of a multigene family) (Morris et al. 1998), *PR*-*2* (a β-1-3-glucanase) (Kauffmann et al. 1987), *PR4* (a chitinase family) (Nasser et al. 1988). Transcriptome studies of *PR* genes with *Azospirillum* sp. B510 applied as inoculum to rice (*O. sativa* L.) reported that one gene was up- and five were down-regulated (Drogue et al. 2014). In another study with *Arabidopsis thaliana* inoculated with *A. brasilense* Sp245, *PR* genes were also up-regulated (Spaepen et al. 2014). In our study, seed inoculation resulted in significant up-regulation of only one *PR* gene in roots, *PR1*, while foliar application in general resulted in up-regulation of *PR1*, *prp2* and *prp4* genes on roots. Up-regulation of *PR1* and *prp4* was also verified with metabolite spray. In relation to the gene expression in leaves, emphasis should be given to single-seed inoculation with both strains that up-regulated all *PR* genes. Interestingly, it has been shown that the use of more than one microorganism optimized ISR responses in pigeon pea (*Cajanus cajan*) (Dutta et al. 2008), similarly to our results with seed inoculation of Ab-V5 + Ab-V6 on roots. *Bacillus subtilis* also up-regulated *PR1* and *PR4*, but not *SOD2* genes in maize roots (Gond et al. 2015). It is also worth mentioning that ISR responses in different tissues from those where the microorganism is applied occurs, e.g. leaf spray with *Pseudomonas fluorescens* in rice induced ISR against the soil-borne plant pathogen *Rhizoctonia solani* (Vidhyasekaran and Muthamilan 1999).

ISR responses to a variety of plant pathogens usually have been associated with the signaling compounds jasmonate and ethylene (Glick 2012; Ahemad and Kibret 2014), the levels of which are increased in tissue independent of SA (Van Loon 2007); this mechanism has also been reported in the association of *Azospirillum* sp. B510 with rice (Yasuda et al. 2009). Indeed, several studies have demonstrated that exogenous applications of SA (Bari and Jones 2009) and JA (Agrawal et al. 2000; Lorenzo and Solano 2005; Wasternack 2007; Bari and Jones 2009) induce *PR* genes and consequently increase the resistance to several pathogens. In addition, exogenous applications of JA also increase the activities of *CAT* and *SOD* enzymes in soybean [*Glycine max* (L.) Merr.] plants stressed by cadmium (Noriega et al. 2012). The ISR might be related also to the reported effects of *A. brasilense* against soil-borne plant pathogens such as *Rhizoctonia* spp. (Russo et al. 2008) and *Fusarium oxysporum* f. sp. *matthiolae* (Somers et al. 2005).

It is worth considering that the exogenous application of synthetic growth regulators (e.g. IAA, GA, kin) has been broadly adopted by foliar spraying due to plant-growth promotion (Halmann 1990), but the commercial products are usually very expensive. However, in our study, the foliar spray of *Azospirillum* metabolites in general improved not only plant growth, but also up-regulated plant genes related to defense mechanisms, and might represent an alternative biological plant regulator.

In conclusion, we reported that, regardless of the method of inoculation—on seeds or by foliar application—the *A. brasilense* strains Ab-V5 and Ab-V6 promoted plant growth. Intriguingly, the foliar application of their metabolites also improved growth. The benefits of cell and metabolite application can be attributed both to the synthesis of phytohormones and to the induction of plant defense-related genes. Clearly, the application of biological low-cost inoculants containing *Azospirillum* cells or their metabolites, promoting plant growth and eliciting plant resistance to biotic and abiotic stresses, have important agronomic implications.
